# Relation between soft tissue energy dissipation and leg stiffness in running at different step frequencies

**DOI:** 10.1098/rsos.231736

**Published:** 2024-06-12

**Authors:** Arthur H Dewolf, André Ivaniski-Mello, Leonardo Alexandre Peyré-Tartaruga, Raphael M. Mesquita

**Affiliations:** ^1^ Laboratory of Biomechanics and Physiology of Locomotion, Institute of NeuroScience, Université Catholique de Louvain, Louvain-la-Neuve, Belgium; ^2^ LaBiodin Biodynamics Laboratory, School of Physical Education, Physiotherapy and Dance, Universidade Federal do Rio Grande do Sul, Porto Alegre, Brazil; ^3^ Human Locomotion Laboratory (LocoLab), Department of Public Health, Experimental Medicine and Forensic Sciences, University of Pavia, Pavia, Italy

**Keywords:** soft tissue, spring-mass-actuator system, running mechanics, biomechanics of running, muscle work

## Abstract

This study aims to investigate the relationship between soft tissue energy dissipation and leg stiffness during running. Eight recreational healthy male runners (age: 22.2 ± 1.0 years; height: 1.84 ± 0.03 m; mass: 73.7 ± 5.7 kg) were asked to run at different speeds and step frequencies. Their soft tissue energy dissipation was estimated by the difference between the total mechanical work of the body, measured as the work done to move the body centre of mass relative to the surroundings plus the work to move the limbs relative to the body centre of mass, and lower-limb joint work. A mass-spring model with an actuator was used to analyse the force–length curve of the bouncing mechanism of running. In this way, the stiffness and damping coefficient were assessed at each speed and step frequency. Pearson's correlations were used to describe the relationship between the deviation from the spring-mass model and soft tissue energy fluctuations. The soft tissue dissipation was found to be significantly influenced by step frequency, with both positive and negative work phases decreasing when step frequency increases. Moreover, deviation from a spring-mass model was positively associated with the amount of soft tissue dissipation (*r* > 0.6). The findings emphasize the substantial role of soft tissues in dissipating or returning energy during running, behaving in a damped-elastic manner. Also, we introduce a novel approach for evaluating the elastic rebound of the body during running. The insights gained may have broad implications for assessing running mechanics, with potential applications in various contexts.

## 1. Introduction

During running, humans must perform mechanical work via the muscle-tendon units to decelerate and then reaccelerate the body centre of mass (CoM) [1]. In a bouncing gait such as running, the positive mechanical work can be provided either by the contraction of the muscle fibres (converting metabolic energy into mechanical energy), or through an elastic energy storage and release mechanism. Indeed, part of the energy of the body can potentially be saved as energy in the elastic tissues of the body (as tendons and ligaments), to be subsequently re-used to propel the body forward and upward.

Based on this energy-saving mechanism, a spring-mass model has been suggested as a simple biomechanical model that successfully explains certain characteristics of running. An example, among the different metrics explained by this model, is the spring stiffness, which is considered to be representative of the capacity of the locomotor system to store and release elastic energy [2–4]. Traditionally, the vertical stiffness is described as the linear relationship formed between the vertical acceleration and vertical displacement of the CoM over the contact phase [5]. The vertical stiffness increases at higher running speeds with self-selected step frequencies (SFs) [3,5–7] and also increases as a function of step frequency at a fixed running speed [8]. Furthermore, the vertical stiffness appears to be more related to a specific step frequency rather than the selected running speed [3].

The linear vertical acceleration–displacement relationship is applicable in steady-state situations (situations where there is as much work being done to sustain the braking and propulsive movements of the CoM), for example when running at constant speed on level ground. In unsteady-state situations, i.e. running on an uphill slope or with a horizontal traction force [9–11], one must do more positive work to raise and accelerate the CoM than negative work to lower and decelerate CoM. In this second scenario, an imbalance in the amount of work produced during the braking and propulsive phases of the CoM exists, and the vertical acceleration–displacement relationship is no longer described as a linear relationship [12]. In slope running, an adapted spring-mass model has been introduced to further describe the movements of the spring by adding an actuator/damper acting in parallel throughout the compression and decompression phases [12,13].

Recently, it has been shown that the mechanical energy fluctuations due to soft tissue energy dissipation (heel pad and foot arch compressions, visceral sway, cartilage and intervertebral disc compressions, etc.) can also affect the mechanical work production in locomotion [14–16]. During running, the body's soft tissues deform and dissipate a fraction of the mechanical energy (negative mechanical work) which is not stored in the elastic muscle-tendon unit. The muscles must actively reset this dissipation to maintain a constant running speed (positive mechanical work) [14]. As a result, running at a constant speed is not a situation where there is as much work being done to sustain the braking and propulsive movements of the CoM; this adaption should be accounted for, and the estimation of the leg-spring stiffness should be updated.

The energy dissipated by the soft tissues has been estimated by comparing different methods used to calculate the mechanical energy output of the body [17]. While different methods exist to estimate the amount of work being done by the muscle-tendon unit, the two methods used here have been widely used in the past to describe the biomechanics of human-legged locomotion [18,19]. The first method is the analysis of the energy changes done to sustain the movements of the CoM against the environment (*E*_com_) and those done to sustain the movements of the limbs relative to the CoM (*E*_int_) (*W*_tot_ = *E*_com_ + *E*_int_) (Willems *et al*., [20]). Whereas a second method focuses on the measurement of the muscular work around the joints (*E_j_*) calculated by the inverse dynamic method [21,22]. *W*_tot_ measures both the rigid and soft tissue movements, while *E*_j_ measures only the rigid-body movements [23]. Therefore, the difference between both methods represents the amount of energy being dissipated by the soft tissues [14,23,24].

In running at 3 m s^−1^ and at the preferred step frequency, the amount of soft tissue energy dissipation accounts for 27% of the total negative work done during the stance phase [15]. Furthermore, the amount and distribution of the negative work done between the active muscle and passive dissipation during a task may be subject dependent and so reflect an individual control of the situation. For example, when landing from a jump, the distribution of negative work done is different when the subjects are instructed to land softly or stiff-legged, the latter reducing the proportion of work done by the active muscles [24]. Based on this, one may expect that a stiffer or softer rebound during running may significantly affect the amount of soft tissue energy dissipation and, thus, the elastic bounce of the body.

Tuning the step frequency at a fixed speed modifies the stiffness of the lower limb [3] and may therefore alter the dissipation of energy by the soft tissues. In this study, we evaluated the amount of passive and active muscular work during running at different step frequencies. In addition, we tested if a relationship exists between the leg stiffness and the amount of soft tissue energy dissipation during running at different step frequencies. The former is estimated based on a projected force-limb compression spring [25,26] with the addition of a damper/actuator to consider the difference between active positive and negative work. Our hypothesis was that an adapted spring system is associated with the amount of mechanical energy dissipation by the body soft tissues, i.e. when a runner increases its limb stiffness, the soft tissue energy dissipations are greater.

## 2. Methods

### 2.1. Participants and experimental procedure

The experimental protocol for this paper was the same as in Mesquita *et al*. [3]; therefore, similar sections will only be briefly explained here. Eight recreational healthy male runners (age: 22.2 ± 1.0 years; height: 1.84 ± 0.03 m; mass: 73.7 ± 5.7 kg) with no injuries in the last six months and able to run 10 kilometres in a single run participated in the study. Informed written consent was obtained and the study followed the guidelines of the Declaration of Helsinki. All procedures were accepted by the UCLouvain ethical committee (B403201838331). A 1-hour training session was done at least 2 days prior to recordings. Subjects that were able to run at all the imposed frequencies were included in the study. A warm-up was proposed voluntarily to each subject prior to the beginning of recordings. For each running condition, the treadmill belt accelerated until the target speed was reached. After an auditory check (each foot strike should correspond to a beat of the metronome) by the experimenters ensuring that the subject was running approximately at the correct step frequency, the recording began and lasted 10 s to register at least 10 strides as in [12]. The belt then decelerated, and in total, each running condition lasted approximately 1 min. Appropriate rest was accorded to the subjects between recording sessions and experimental conditions were randomized to avoid habituation and fatigue bias.

The number of subjects chosen was based on an *a priori* statistical power analysis (Power: 1 − ß = 0.8, minimal sample size = 8; GLIMMPSE 3.1.2 Denver Colorado, USA) where ß is the probability of making type II error, from results (effect of frequency on vertical stiffness) of a pilot study [27]. Participants ran on a treadmill at four different constant speeds: 8, 11, 14 and 17 km h^−1^. At each speed, subjects were asked to run at 5 imposed step frequencies: 2.0, 2.4, 2.8, 3.2, 3.6 step s^−1^ conveyed via an electronic metronome.

### 2.2. Experimental set-up

Participants ran on an instrumented treadmill (h/p/Cosmos, Germany—Arsalis, Belgium) with a belt surface of 1.6 × 0.65 m. The entire treadmill was mounted onto four strain-gauge force transducers that measure the three components of the ground reaction force exerted under the foot by the treadmill [25]. The signal, recorded from each transducer at a sampling rate of 1 kHz, was amplified and filtered by a two-way eighth-order Bessel filter with a lowpass cut-off frequency of 10–30 Hz [10].

Bilateral, full-body three-dimensional (3D) kinematics was recorded at 200 Hz by means of a Qualisys system with 13 cameras (12 Mocap Oqus 6 + cameras and one video Miqus M1 camera Qualisys, Sweden) placed around the treadmill. Participants were equipped with 29 retro-reflective markers glued onto the skin at the following positions: chin-neck intersect (neck), sternum (chest), superior surface of acromion (shoulder), lateral epicondyle of humerus (elbow), ulnar styloid process (wrist), superior anterior iliac spine (waist), superior posterior iliac spine (backwaist), greater trochanter (GT), mid-thigh (thigh), external condyle of femur (knee), shin (shank), lateral malleolus (ankle), heel (heel), fifth metatarsophalangeal joint (VM) and second metatarsophalangeal joint (IIM). Kinematic data were then oversampled at 1 kHz using the spline routine in Matlab. The kinematic data was filtered by the same filter with a low-pass cut-off frequency at 30 Hz.

### 2.3. Data processing

Data processing was performed via custom-made programs written with LABVIEW (National Instruments 2019, Austin, Texas, USA) and Matlab software.

#### 2.3.1. Gait separation

A stride period was defined based on the time between a right foot contact and a following right foot contact. A foot contact corresponds to the time at which the vertical component of the ground reaction force (*F*_z_) is greater than 10% of body weight. The step period is defined as the time between a right foot contact and the left foot contact.

#### 2.3.2. Computation of acceleration, velocity and vertical displacement of the centre of mass

The acceleration, velocity and displacement of the CoM was calculated from the ground reaction force (GRF) as in Gosseye *et al*. [28]. Briefly, the fore–aft and vertical CoM accelerations were obtained respectively as $\;a_{f}=F_{y}/m$ and $a_{v}=(F_{z}/m)-g$ , where *F*_y_ is the fore–aft GRF and *g* is the gravitational acceleration. We did not consider the lateral component of the GRF since its contribution is negligible [20].

A time-integration of the *a*_f_ and *a*_v_ recordings give the fore–aft (*v*_f_) and vertical (*v*_v_) velocity changes of the CoM. Integrations are performed numerically by the trapezoidal method. An integration constant must be added to *v*_f_ and *v*_v_ to obtain the components of the instantaneous velocities of the CoM relative to a reference frame fixed to the treadmill as in Gosseye *et al*. [28]. The vertical (*S*_v_) displacement of the CoM relative to the treadmill is then computed by numerical integration of *v*_v_.

#### 2.3.3. Computation of the external work (*E*_com_), work (*W*_ext_)

**Figure 1.**
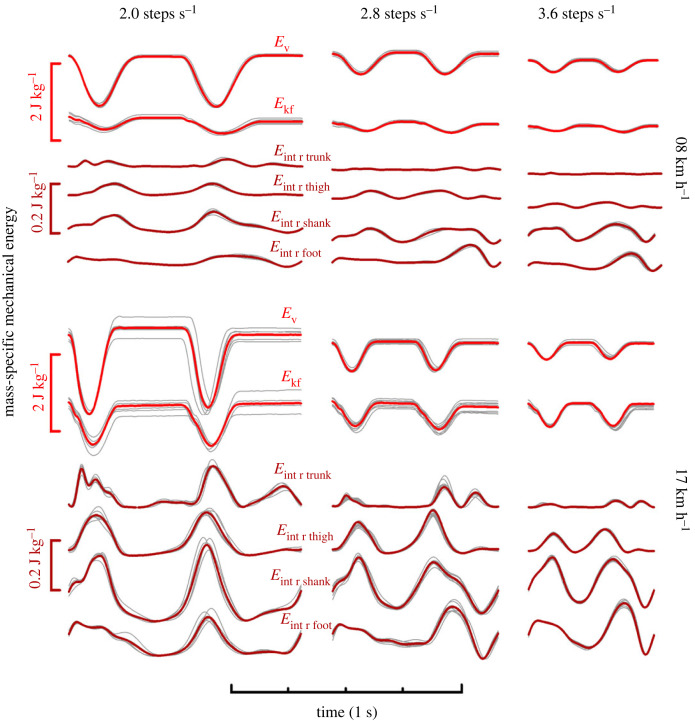
The red (average across stride) and corresponding grey (individual stride) typical time-curves represent respectively, from top to bottom, the normalized energy done to sustain the movements in the vertical direction (*E*_v_) and the energy done to sustain the movements in the fore–aft direction (*E*_kf_), the normalized internal energy of the trunk, right thigh, right shank and right foot relative to the CoM at 2, 2.8 and 3.6 steps s^−1^ at both 08 (top panel) and 17 km h^−1^ (bottom panel).

**Figure 2.**
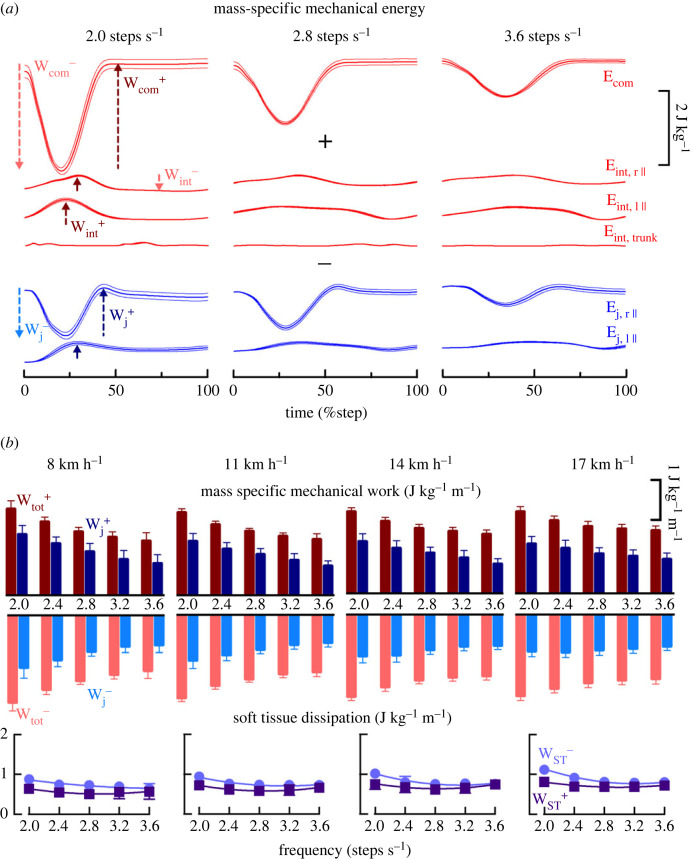
(*a*) The red (average across subjects) and corresponding filled area (standard deviation) time-curves represent, from top to bottom, the normalized energy done to sustain the movements of the centre of mass (*E*_com_), the normalized internal energy of the right lower-limb, of the left lower-limb and of the trunk relative to the CoM at 2, 2.8 and 3.6 steps s^−1^ at 14 km h^−1^. The blue (average across subjects) and corresponding filled area (standard deviation) time-curves represent the joint work (ankle, knee and hip together) of the right (top) and left (bottom) limb. (*b*) Positive (positive values) and negative (negative values) mass-specific mechanical work, computed from *E*_com_ and *E*_int_ (red bars) and from the joint work (blue bars) at the four different speeds and presented as a function of step frequency. The bars represent the grand mean of all the subjects and the thin lines represent one standard deviation. The difference between the two methods corresponds to the positive and negative work done by the soft tissues, presented as a function of speed and frequency in the lower panel. The line which passes through all points is a quadratic nonlinear regression as defined in Graphpad-Prism (Dotmatics, CA, USA).

The energy used to sustain the movements of the CoM (*E*_com_) against the environment was computed as the sum of the energy done to sustain the vertical (*E*_v_) and fore–aft (*E*_kf_) movements. *E*_kf_ can be computed by $E_{\mathrm{kf}}=\int F_{y}\;(v_{f}-V_{\mathrm{avg}})dt=1/2\;m\;{\left(v_{f}-V_{\mathrm{avg}}\right)}^{2}$ , where $V_{\mathrm{avg}}$ is the average speed of the treadmill. *E*_v_ can be computed by $E_{v}=\int (F_{z}\;v_{v})\;\mathrm{dt}=1/2\;m\;v_{v}^{2}+m\;g\;S_{v}$ . Then, the *E*_com_ is given by $E_{\mathrm{com}}\;=\;E_{\mathrm{kf}}+E_{v}$ . The positive/negative external work done to move the CoM relative to the surroundings ( $W_{\mathrm{com}}^{+/-}$ ) is then calculated as the sum of the positive/negative increments from the *E*_com_ curve over one stride (figures 1 and 2).

#### 2.3.4. Computation of the internal work ( $W_{\mathrm{int}}$ )

The computation of the internal work done to accelerate and rotate the limbs relative to the CoM has been described in detail in Willems *et al*. [20]. Both lower limbs were modelled as a multi-segmented limb formed by thigh, shank and foot segments. The internal energy of an *i*th segment is given by $E_{\mathrm{int}}^{i}=1/2\;(m_{i}\;V{{\;}_{i}^{\prime }}^{2}+\;I_{i}\;\omega_{i}^{2})$ where *m*_i_ is the mass of the segment and *I*_i_ is its moment of inertia around its centre of mass, *V*_i_’ is the translational velocity of the segment's centre of mass relative to the CoM and *ω*_i_ is the rotational velocity of the segment. The energy of the segments from the same limbs were added to obtain the energy of each lower limb. This procedure assumes that energy transfers are only possible between segments of the ipsilateral limb, but not with the contralateral ones [20,29]. The positive/negative internal work done per segment, $W_{\mathrm{int},i}^{+/-}$ , is calculated as the positive/negative increments of the internal energy of each lower limb and at the level of the trunk (figure 1). As such, the movements of the arms are not considered in the *W*_int_ calculations. This is considered in detail in the Discussion section below.

#### 2.3.5. Computation of joint work (*W*_j_)

The joints between limb-segments: ankle, knee and hip joints, are simplified as hinge joints implying that each has only one degree of freedom. By combining both kinematic and kinetic variables, the net muscular moment of the lower-limb joints (respectively $M_{j,a}$ , $M_{j,k}$ and $M_{j,h}$ ) were evaluated in the sagittal plane by an inverse dynamic method [21,22].

**Figure 3.**
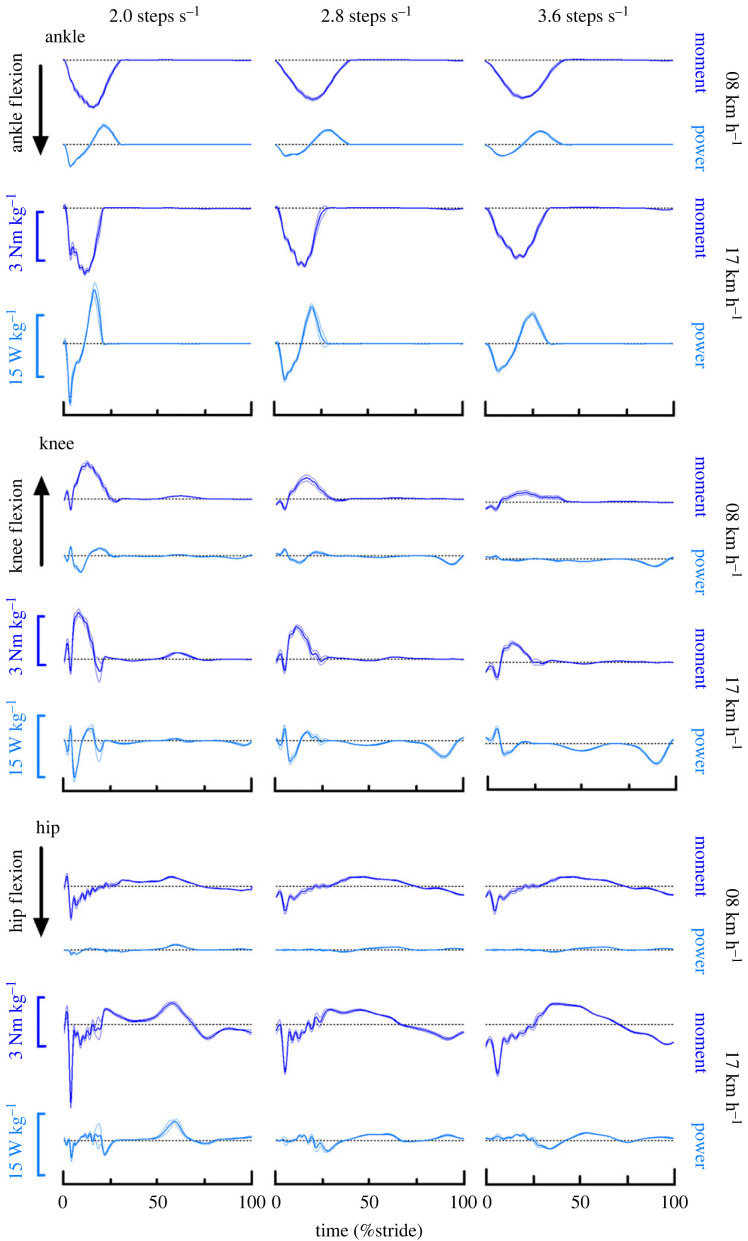
Mean ankle, knee and hip (top, middle and bottom panel, respectively) joint moment and power trajectories over a running stride at 8 and 17 km h^−1^ and at 2 (left), 2.8 (middle) and 3.6 (right) steps s^−1^. Moments and power shown are sagittal plane values. Moments are defined as positive in extension for the ankle and hip, and flexion for the knee.

The mass $m_{s}$ , position of the centre of mass and radius of gyration about its CoM of each segment were the same as that of the internal work. Each segment being in motion equates to $\sum_{i}F_{i}=m_{s}a_{s}$ , where ***F***_*i*_ is the *i*th force applied on the segment and $a_{s}$ is the double time derivative of the segment's centre of mass displacement. The torque generated around each joint can be computed as $\underset{i}{\sum }d_{i}\;x\;F_{i}+\sum_{i}M_{j}=I_{s}\alpha_{s}$ , where ***d*** *_i_* is the lever arm of ***F*** *_i_*, ***M*** *_j_* is the net muscular moment applied on the joint, $I_{s}$ is the moment of inertia of the segment and $\alpha_{s}$ is the angular acceleration of the segment as compared with the horizontal. The net muscular power generated at each joint (*P*_j_) was calculated as $P_{j}=\;M_{\;j\;}\omega_{j}$ . Where $\omega_{j}$ is the time derivative of the angle formed between two segments. As in the computation of internal work, energy transduction between joints must be taken into account [14]. The powers of the joints from the same limb were added to obtain the joint power of each limb, *P_j,i_*. Accordingly, the muscular energy done at joint level per limb (*E_j,i_*), was determined by the time-integration of the summed joint power, $E_{\;j,i}=\int P_{\;j,i}\;dt$ . The positive/negative joint work done at each limb, $W_{\;j,i}^{+/-}$ , is calculated as the sum of the positive/negative increments of this curve (figure 3).

#### 2.3.6. Computation of total work (*W*_tot_) and soft tissue energy dissipation (*W*_ST_)

The total work of the body is calculated by assuming no energy transfer between external and internal energies but considers energy transfer between same-limb segments for the movements of said segments compared with the CoM [20]. Therefore, the positive/negative increments of *W*_tot_ are given by the sum of the *W*_com_ increments and those of *W*_int_ from each limb and at the level of the trunk, $W_{\mathrm{tot}}^{+/-}=\;W_{\mathrm{com}}^{+/-}$ + $W_{\mathrm{int},R}^{+/-}+W_{\mathrm{int},L}^{+/-}+\;W_{\mathrm{int},\mathrm{trunk}}^{+/-}$ . The positive and negative work increments dissipated by soft-tissue deformation (*W*_ST_) is calculated as the difference between the respective positive or negative increments of total work and that of joint work done per limb, $W_{\mathrm{ST}}^{+/-}=\;W_{\mathrm{tot}}^{+/-}-\;W_{j,R}^{+/-}-\;W_{j,L}^{+/-}$ (figure 2).

#### 2.3.7. Projected force, stiffness, damping coefficients and area

The spring-mass characteristics of the leg were assessed by computing the force–length curve during contact using a method adapted from Gill *et al*. [25] and Dewolf *et al*. [12]. The force used in the present model is the force vector projected along the leg (*F*_projected_). To do this, the lower limb angle was calculated as the angle between the line crossing the centre of pressure under the foot measured by the treadmill and the GT marker. The centre of pressure being not reliable for low vertical force values, a 300 N vertical threshold was used.

The projected force was normalized by the mass of the subject. The leg length (*L*_leg_) was defined as the distance between the centre of pressure under the foot and the GT marker. The compression of the leg starts at touchdown until the minimum of *L*_leg_, followed by the leg decompression. The difference between the energy of the two phases were compared. In particular, the area between the compression and decompression phase of the force-displacement curve were estimated as

$$\Delta_{\mathrm{area}}=\int_{\min L_{\mathrm{leg}}}^{\mathrm{end}}F_{\mathrm{projected}}\;dL_{\mathrm{leg}}-\int_{0}^{\min L_{\mathrm{leg}}}F_{\mathrm{projected}}\;dL_{\mathrm{leg}}.$$

To describe the force–length relationship during contact, the classical spring-mass model [5,30] was implemented by adding an actuator parallel to the spring [12]. This actuator generates a force proportional to the velocity of change in leg length (velocity of leg compression-decompression, *V*_leg_),

$$F_{\mathrm{projected}}\;=cst+k\;L_{\mathrm{leg}}\;+c\;V_{\mathrm{leg}},$$

where *cst* is a constant, *k* represents the overall stiffness of the leg and *c* is the actuator coefficient. The coefficient *c* is negative when the actuator works like a damper absorbing energy.

### 2.4. Statistics

For each participant, the parameters were averaged first across the right and left steps for each stride then averaged per stride. Then descriptive statistical analysis was performed. First, the Shapiro–Wilk test was performed to verify normality. When the data did not meet the normal distribution criteria (Shapiro–Wilk's W-test, *p* < 0.05) non-parametric statistics were used for data analysis. A generalized linear mixed effect model with Bonferroni *post hoc* correction was used to assess the individual and interaction effects of frequency and speed on the calculated variables ( $W_{\mathrm{tot}}^{+/-}$ , $W_{j}^{+/-}$ , $\;W_{\mathrm{ST}}^{+/-}$ , *k*, *c* and Δ_area_). Statistical tests were run on IBM SPSS Statistics (PASW Statistics, 19, SPSS, IBM, Armonk, NY, USA). Pearson's correlation coefficients (*r*) were used to quantify the relationship between variables (*k*, *c,* $W_{\mathrm{ST}}^{+/-}$ and Δ_area_). The results of the statistical tests were considered significant for a *p*-value less than 0.05. When a curve was drawn on figures, these were based on quadratic fits done on GraphPad Prism (GraphPad Software, LLC, Dotmatics, San Diego, CA, USA).

## 3. Results

### 3.1. Soft tissue energy dissipation

As the subjects are running at constant speeds over each trial, there is as much mechanical work done to sustain the movements of the CoM during the braking and propulsive phases. Therefore, the positive and negative total mechanical work of the body ( $W_{\mathrm{tot}}^{+}$ and $W_{\mathrm{tot}}^{-}$ ) were equal. Both $W_{\mathrm{tot}}^{+}$ and $W_{\mathrm{tot}}^{-}$ did not change with speed ( $W_{\mathrm{tot}}^{+}$ : *F* = 2.58, *p* = 0.07, $W_{\mathrm{tot}}^{-}$ : *F* = 2.96, *p* = 0.52) but decreased as SF increased ( $W_{\mathrm{tot}}^{+}$ : *F* = 153.41, *p* < 0.001, $W_{\mathrm{tot}}^{-}$ : *F* = 160.09, *p* < 0.001).

Likewise, when considering the joint work, both $W_{j}^{+}$ and $W_{j}^{-}$ had similar speed and frequency effects: $W_{j}^{+}$ and $W_{j}^{-}$ decreased as speed ( $W_{j}^{+}$ : *F* = 10.55, *p* < 0.001, $W_{j}^{-}$ : *F* = 10.75, *p* < 0.001) and SF increase ( $W_{j}^{+}$ : *F* = 86.99, *p* < 0.001, $W_{j}^{-}$ : *F* = 28.32, *p* < 0.001).

Soft tissue energy dissipation was measured by considering the difference between $W_{\mathrm{tot}}^{+/-}$ and $W_{j}^{+/-}$ . Regarding the negative work phase, the amount of energy dissipation increased as a function of speed (*F* = 17,52 *p* = 0.027) and decreased as a function of SF (*F* = 26.25, *p* < 0.001). The amount of energy dissipation occurring during the positive work phase increased as a function of speed (*F* = 23.35, *p* < 0.001) and increased as one deviates from 2.8 steps s^− 1^ both when running at lower and higher SFs (*F* = 7.13, *p* < 0.001) (figure 2).

### 3.2. Bouncing mechanism of running

**Figure 4.**
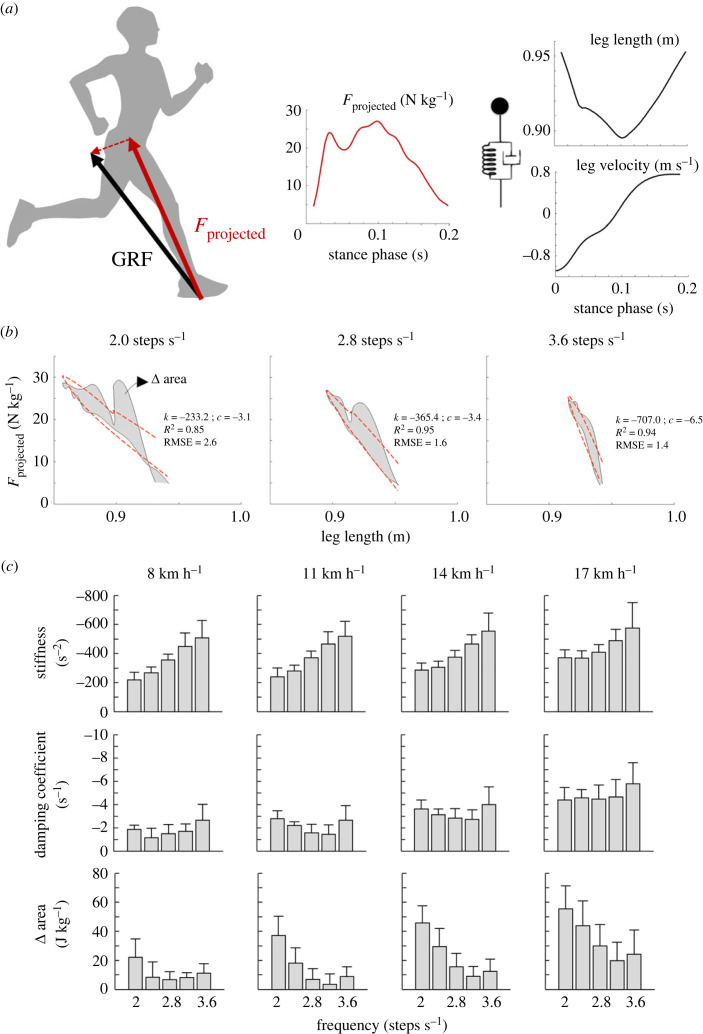
(*a*) Schematic representation of the method used to compute leg stiffness. On the left, one typical trace of the force projected on the leg, the leg length and leg velocity during on typical strep (same subject as figure 1). The insets illustrate the model used. (*b*) Typical trace of the projected force during the contact period plotted as a function of leg length during running at 2, 2.8 and 3.6 steps s^−1^ at 14 km h^−1^. The red dashed lines correspond to the predicted value of *F*_projected_ computed using the values of *b*, *k* and *c* obtained by the regression analysis. The *R* ^2^ and RMSE values are indicated on each trace. (*c*) Stiffness, damping coefficient and the area between the compression and decompression phase of the force-displacement curve at the four different speeds and presented as a function of step frequency. The bars represent the grand mean of all the subjects and the thin lines represent one standard deviation.

During running, the leg can be modelled as a spring-mass mounted in parallel with an actuator that generates a muscular force proportional to *V*_leg_. The linear regression model demonstrated a strong fit to the data, as indicated by an average RMSE across all the steps of 1.44 ± 0.64 N kg^−1^. Furthermore, the model's ability to explain the variation in the dependent variable was substantial, as evidenced by an average *R*² value of 0.95 ± 0.04 (figure 4 *b*).

The overall mass-specific stiffness *k* and the actuator coefficient *c* generated by the lower-limb muscles (figure 4 *c*) increased with both the SF (*k*: *F* = 46.1, *p* < 0.001; *c*: *F* = 6.3, *p* < 0.001) and the speed of progression (*k*: *F* = 10.7, *p* < 0.001; *c*: *F* = 68.7, *p* < 0.001). The difference of area (Δ_area_) under the force–length curve during compression and decompression was also affected by speed (*F* = 30.2, *p* < 0.001) and frequency (*F* = 27.4, *p* < 0.001): the area was greater at fast running speed and at low step frequencies (figure 4 *c*).

**Figure 5.**
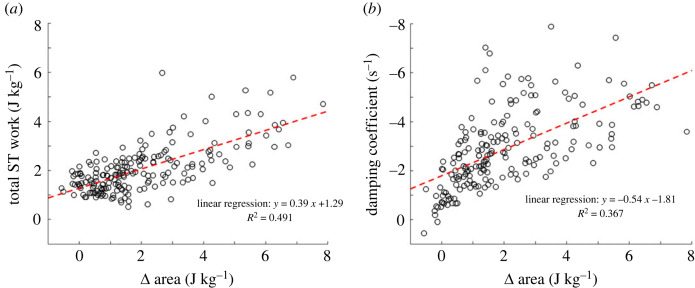
Correlations between the area between the compression and decompression phase of the force–displacement curve (normalized by body weight) and other gait parameters. Each point represents the stride-averaged value for one subject. Linear regression lines are also plotted. (*a*) Relationships between the area and the total soft tissue (ST) work (positive + negative). (*b*) Relationships between the area and the damping coefficient of the damped spring-mass model. The *R* ^2^ values are indicated on each graph.

The changes in soft tissues energy dissipation and return ( $W_{\mathrm{ST}}^{+/-}$ ) were correlated with the changes in the Δ_area_ (figure 5). The correlation coefficient of the linear regression between the two was *r*_(191)_ = 0.70 (95% CI [0.62, 0.76]); *p* < 0.001). Also, the damping coefficient *c* was correlated with the changes in the Δ_area_, with a correlation coefficient of *r*_(191)_ = 0.61 (95% CI [0.51, 0.69]); *p* < 0.001). Instead, the stiffness *k* was not significantly correlated with the amount of soft tissues dissipation (*r*_(191)_ = 0.13; *p* = 0.071).

## 4. Discussion

The present paper aims at investigating the relationship between the soft tissue energy fluctuation and the stiffness of a lower limb spring-mass-actuator system during running at different speeds and step frequencies. As already observed during landing from a jump [24], changing the running frequency, and in turn the lower-limb stiffness [3,5,8,31] (figure 4), significantly affects the amount of soft tissue energy dissipation and elastic return (figure 2). In addition, a linear relationship was observed between the deviation from a spring-mass model (evaluated by the area between the force–length curve during compression and decompression, or by the damper coefficient; figure 4 *b*) and the soft tissue positive and negative work (*r* > 0.61). That is, when there is a greater loss of energy during the contact phase between the elastic energy storage (compression) and release (decompression), a greater energy dissipation and return by the soft tissues of the body is also observed.

Our results show that soft tissues performed significant negative and positive work during the stance phase (figure 2). At all speeds and frequencies, more negative than positive work is performed, supporting the idea that soft tissues may behave in a damped elastic manner [15]. In addition, consistent with previous findings [14,24], greater soft-tissue energy dissipation occurs in human movement when the leg impacts the ground (termed the collision) during walking [32] and during running [14]. Furthermore, a stiffer landing strategy results in a greater amount of soft tissues dissipation [24]. Here, we observed higher dissipation at faster speeds and at low frequencies (figure 2), when the impact with ground is higher [3,33].

### 4.1. Comparison with prior literature and methods

The measurement of mechanical work (figure 2) was in good agreement with previous literature reporting *W*_com_ and *W*_int_ [3,34] and lower-limb joint work [35,36] during running at different step frequencies. The estimate of the substantial soft tissue network suggests that ‘traditional’ methods (figures 1 and 3) may either over- or under-estimate the muscular work performed by the body during running. The present approach may help to refine a more accurate model for estimating the contribution of mechanical muscle cost, as proposed by Riddick & Kuo [14]. However, certain methodological details need to be discussed in order to accurately determine active and passive contributions.

Zelik and Kuo [16] marked the first endeavour to estimate soft tissue work indirectly during walking. In their paper, the authors used the difference between rigid-body joint work from inverse dynamics and *W*_com_ (excluding *W*_int_) as an indicator of soft tissue deformations. In the latest study on soft tissue work by the same research group [37], both lower-limb and upper-limb joint powers and *W*_int_ were considered. Recently, Riddick & Kuo [14] adopted a similar approach, albeit without including the upper limb. We opted to follow the approach of the latter authors [14] due to the minor power contributions of arm movement during running [15]. Additionally, lower-limb joint powers were estimated using a bottom-up approach [38], while upper-limb joint kinetics were computed using a top-down approach, similar to the computation of *W*_int_. Therefore, the inclusion (or exclusion) of arm movements would not significantly alter our estimation of soft tissue work. In the end, the segments included are the trunk, thigh, shank and foot, required to compute the hip, knee and ankle joint angles.

Both Riddick & Kuo [14] and Honert & Zelik [37] compared joint work with *W*_tot_ considering energy transfers between all (ipsilateral and contralateral) segments for the calculation of *W*_int_ and allowing transfers between *W*_int_ and *W*_com_. As discussed in Willems *et al*. [28], permitting energy transfer between all limb segments results in a 20% reduction in the total work amount. Moreover, these authors argue that considering energy transfer between limb segment energy and CoM energy without accounting for the feasibility of such transfer is rather complex. While such transfers may potentially be plausible [5], quantification is necessary to refine our measurement of soft tissue energy fluctuation. Here, we assumed transfer between ipsilateral body segments (thigh, shank, foot) for internal work measurement and between ipsilateral joints (hip, knee, ankle) for joint work measurement. This method appears more suitable for running, where transfer between legs is less likely than during the double support phase of walking [39].

### 4.2. Relation with the bouncing mechanism of running

The discrepancy between negative and positive soft tissues energy may also have implications for the mechanics of running. As running speed increases, an increase in the area (hysteresis) between the force–length curve during compression and decompression can be observed (figure 4 *a*), suggesting a deviation from a linear elastic behaviour. This deviation from the simple spring-mass model presumes that part of the work must be performed actively by the muscle. Therefore, energy dissipation from soft tissues can account for a significant amount of metabolic cost [14] and should be considered when studying running energetics. Still, including measurements of tendon contributions in the recovery of elastic energy from muscle–tendon structures would be required to further understand if the deviation from a spring-mass model observed here reflects a change in the mechanical behaviour of the muscle-tendon units (MTUs) [40,41]. Also, the relative contribution of tendon length changes to MTU length changes will most likely have a stronger association with stiffness rather than the dissipation of soft tissue energy dissipation. Indeed, a study of the *in vivo* Achilles tendon mechanical behaviour during hopping at different frequencies suggests that the tendon hysteresis decreases with increasing frequency and that the contributions of the elastic elements to overall mechanical power reaches a maximum around 3 Hz [41]. Interestingly, the same trend can be observed on Δ area (figure 4 *c*), with a minimum difference in the work between compression and decompression phases (closer to a linear elastic behaviour) occurring at approximately 3.2 Hz (considering all running speeds).

The deviation from the elastic spring is confirmed by the greater damping coefficient at fast speeds. Also, at higher step frequency, together with the already documented increase in lower-limb stiffness, a reduction of the hysteresis supports the optimization of the elastic mechanism documented by Mesquita *et al*. [3]. Interestingly, the area between the force–length curve during compression and decompression is positively associated with the total soft tissue work dissipation, indicating that the deviation from an elastic spring can be partly related to the energy fluctuation of the soft tissues (figure 5).

The use of the updated methods of evaluating the bouncing mechanism of running to quantitatively assess the stiffness and damping coefficient proposed here may open new areas of investigation of muscle physiology during running. Indeed, it may help to monitor running mechanics in different types of pathologies, after immobilization, training routines, etc.

### 4.3. Limitations

The approaches used to compute MTU work are not the only methods for calculating mechanical work in running (e.g. [11,32,42,43]). The two methods used here are the most used to relate metabolic energy expenditure and mechanical work [18]. Still, there are several assumptions/limitations and differences to acknowledge.

The separate calculation and final summation of *W*_int_ and *W*_com_ could overestimate *W*_tot_ [18]. Also, there are many other types of internal work not considered, such as the work needed to overcome internal friction among joints and tissues [44]. Moreover, the inverse dynamics used to measure *W*_j_ and *W*_int_ rely on assumptions for the inertial parameters (segmental mass, inertia, kinematics, and CoM and joint centre location). Also, *W*_j_ assumes that the body is made of rigid-body segments and, thus, does not consider work of non-rigid segment deformations—for example, due to passive wobbling of viscera, shoe cushion, fat tissue vibration, etc. The *W*_tot_ instead captures contributions from muscles and tendon, but also other soft tissues in the body.

The comparison of both methods revealed that they both methods agree strongly, suggesting that they do reflect the primary contributors to movement. By comparing one versus the other, we can estimate the deviation from rigid-body segments, occurring mainly following an impact/collision with the ground [24,45], and that has been related to the estimated soft tissues deformation [14,23]. Soft tissue motion occurs during movement, and the estimation of soft tissue energy dissipation used here does not fully capture the complexity of soft tissue behaviour. For example, the effect of inertial and elastic properties of these tissues on the dynamics of movement is still largely unknown [46] and requires further investigation.

## Data Availability

Zenodo repository to ensure easy access to our data: (https://zenodo.org/records/10598063) [47]. Supplementary material is available online [48].
